# Mutations at Positions 181 and 185 Within the LCMV GP Protein Increase Tumor Cell Infectivity, Limit Induction of Type I Interferon, and Accelerate T Cell Activation

**DOI:** 10.3390/v18091002

**Published:** 2026-09-11

**Authors:** Michael Bergerhausen, Lisa Holnsteiner, Sarah-Kim Friedrich-Becker, Zhongwen Hu, Rosa Schmitz, Dethardt Müller, Marcus Kostka, Tim Brandenburg, Haifeng C. Xu, Cornelia Hardt, Jörg Vollmer, Philipp A. Lang, Karl Sebastian Lang

**Affiliations:** 1 Abalos Therapeutics, 40225 Düsseldorf, Germany; bergerhausen@abalos-tx.com (M.B.); friedrich@abalos-tx.com (S.-K.F.-B.); mueller@abalos-tx.com (D.M.); kostka@abalos-tx.com (M.K.); vollmer@abalos-tx.com (J.V.); 2 Institute of Immunology, University Hospital Essen, 45147 Essen, Germany; huzhongwen9@gmail.com (Z.H.); tim.brandenburg@uk-essen.de (T.B.); conny.ynnoc@t-online.de (C.H.); karlsebastian.lang@uk-essen.de (K.S.L.); 3 Department of Molecular Medicine II, Medical Faculty, Heinrich Heine University, 40225 Düsseldorf, Germany; xuh@hhu.de (H.C.X.); langp@uni-duesseldorf.de (P.A.L.)

**Keywords:** LCMV, type I interferon, CD8^+^ T cells, viral tropism, innate immunity, tumor tropism

## Abstract

The lymphocytic choriomeningitis virus (LCMV) represents a strong T cell activating virus with anti-tumoral properties. In recent work, we developed an attenuated cancer cell-adapted reassortant LCMV strain carrying tumor-tropic mutations. The immunological mechanisms underlying these mutations remained unknown. Here, we passaged wild-type LCMV-WE in human lung cancer H1975 cells for 52 passages. After 29 passages, the virus acquired the GP1 mutations I181M and R185W, involved in viral receptor interactions, which remained stable throughout the subsequent 33 passages. Compared with wild-type LCMV-WE, LCMV-P52 showed increased infectivity, partly concomitant with enhanced propagation in human tumor cells. In vivo, LCMV-P52 induced reduced type I interferon (IFN-I) responses and showed accelerated expansion in CD169^+^ macrophages. Propagation within the splenic marginal zone correlated with accelerated priming of virus-specific CD8^+^ T cells and limited liver tissue damage. Mechanistic in vitro experiments showed that the I181M and R185W mutations were associated with substantially reduced infection of plasmacytoid dendritic cells (pDCs). Collectively, the I181M and R185W mutations limit systemic IFN-I induction and accelerate LCMV propagation in CD169^+^ macrophages, correlating with improved CD8^+^ T cell function and reduced liver damage. These findings provide mechanistic insight into how defined GP mutations can modulate viral tropism and antiviral immune responses.

## 1. Introduction

The lymphocytic choriomeningitis virus (LCMV) is a small, single-stranded RNA virus from the *Mammarenavirus* family. Its natural host is *Mus musculus*, and it is widely used to study fundamental immunological mechanisms. Over the past decades, several immunological concepts have been identified using LCMV infections in mice. Depending on the LCMV strain, infection outcomes can differ substantially. For example, the Armstrong and WE strains induce strong T cell responses, resulting in acute infections that are typically controlled within 6–20 days [1]. In contrast, strains like Docile and Clone 13 drive T cell exhaustion, leading to viral persistence for more than 100 days [1]. Host mechanisms that modulate T cell responses during infection are well described. However, little is known about how viral mutations interact with host cells and thereby alter innate and adaptive immune activation, ultimately shaping the outcome of infection. In our previous study, we identified the naturally acquired GP mutations I181M and R185W as key components of an optimized tumor-tropic arenavirus with enhanced anti-tumor efficacy [2]. Although the therapeutic potential of this optimized virus was established, the mechanistic contribution of these mutations to viral tropism and immune activation remains unresolved.

The early phase of the immune response is characterized by fast replication of LCMV in antigen-presenting cells (APCs) [3]. This replication depends on an early IFN-I response and the expression of endogenous interferon inhibitor USP18 [3,4,5]. Viral replication after infection leads to the production of viral antigens and ligands for pattern recognition receptors. Within endosomes, viral RNA is recognized by TLR7 and TLR8 at an early timepoint. In the cytoplasm, RIG-I and MDA5 detect intracellular RNA species of replicating viruses, leading to increased serum levels of IFN-α [6]. In addition to the recognition of RNA, TLR2 plays a role in the induction of IL-6 and monocyte chemotactic protein-1 (MCP-1) in mice [7] and in isolated glial cells [8] when infected with LCMV-Armstrong. Plasmacytoid dendritic cells (pDCs) are professional IFN-I-producing cells and are of major importance for the early IFN-I response during virus infection [9]. Due to their high expression of IRF3 and IRF7, they can quickly produce high levels of IFN-I after uptake and/or infection. Production of virus antigens within APCs is essential for fast priming of a robust antiviral CD8^+^ T cell response [3]. In contrast, strong IFN-I responses and early viral suppression can limit CD8^+^ T cell expansion and promote their exhaustion. In addition, regulatory signals like IFN-I modulate DC survival as well as IL-10 and PD-L1 induction, thereby shaping the cytotoxic effector and memory T cell functions [10,11].

By applying different LCMV strains and modulating viral titers the initial replication in APCs, innate immune activation and CD8^+^ T cell priming can be differentially activated [10,11,12]. In this interaction, IFN-I plays an essential role [10,11]. While it is well established that low infection doses allow for prolonged viral propagation in APCs, the role of different viral factors for the replication in APCs remains to be studied.

Our previous study demonstrated that the optimized reassortant virus WE-CL13-GP181M-185W-492I exhibited enhanced propagation in multiple human tumor cell lines together with potent T cell-dependent anti-tumoral efficacy and a favorable preclinical safety profile [2]. Building on these findings, the present study investigates the mechanistic contribution of the naturally acquired GP mutations I181M and R185W to viral tropism, innate immune activation, and antiviral T cell responses. In a companion study entitled “*Lymphocytic choriomeningitis virus bearing GPC mutations for enhanced tumor tropism and strong anti-tumor activity*” [13], we demonstrated that incorporation of the GP I181M and R185W mutations into a recombinant LCMV vector enhances tumor tropism and therapeutic efficacy in multiple preclinical tumor models. The present study provides the mechanistic basis for these observations by elucidating the molecular and immunological mechanisms underlying the phenotype of these mutations. A preliminary version of parts of this work has previously been included in the doctoral dissertation of Michael Bergerhausen [14].

Here, we aimed to introduce specific point mutations into the wild-type LCMV-WE strain and analyze their impact on IFN-I induction and T cell activation. In these experiments, we identified two mutations at positions 181 and 185 of the viral GP protein. When analyzing the role of these mutations in T cell responses, we found that the combination of both mutations accelerated priming of CD8^+^ T cells. Collectively, our data indicate that the mutations are associated with reduced pDC infection and limited systemic IFN-I induction. Therefore, systemic IFN-I induction was delayed which correlated with accelerated viral replication in APCs.

## 2. Materials and Methods

### 2.1. Cell Lines

To obtain complete growth medium (CGM), DMEM (PAN Biotech GmbH, Aidenbach, Germany, P04-03600) was supplemented with 10% FCS (Gibco, Grand Island, NY, USA) for Gist-T1, MaMel57, MaMel86a, and SW872 (HTB-92) or 5% FCS for MC57G and BHK-21 (CCL-10) cells. For H1975 (CRL-5908) and HCC1954 (CRL-2338), RPMI-1640 (Pan Biotech, P04-18047) supplemented with 10% FCS was used as CGM. All media were additionally supplemented with 100 U/mL penicillin, 100 µg/mL streptomycin and 292 µg/mL L-glutamine (Sigma-Aldrich, St. Louis, MO, USA, G6784). Gist-T1 cell line was provided by Prof. S. Bauer (University Hospital Essen, Germany). MaMel51 and MaMel86a were obtained from Prof. A. Paschen (University Hospital Essen, Germany). MC57G cells were donated by Prof. R. Zinkernagel (University of Zurich, Switzerland). All other cell lines were initially purchased from ATCC (Manassas, VA, USA). Neuronal progenitor cells (Neurons), provided by Prof. J. Gopalakrishnan (University Hospital Düsseldorf, Germany), were differentiated using the STEMdiff Forebrain Neuron Differentiation and Maturation Kit (Stemcell Technologies, Vancouver, BC, Canada, 08600).

### 2.2. Virus Passage

Virus passage was performed by repeated infection of human lung carcinoma cells H1975. Therefore, 2 × 10^5^ cells were seeded in 1 mL CGM into a well of a 24-well plate and infected with virus. For the first passage, cells were infected with the LCMV-WE strain with an MOI of 1. After 72 h, 10 µL of the virus-containing supernatant was transferred to the next well with again 2 × 10^5^ naïve H1975 cells. The remaining supernatant was stored at −80 °C for later analysis. These steps were repeated for each passage.

### 2.3. Virus Stock Generation

For virus stock production, BHK-21 cells were cultured in a T175 flask to 60–70% confluence in CGM. For initial infection, medium was removed from adherent cells and replaced by 5 mL DMEM + 2% FCS, containing 2 × 10^4^ FFU of desired virus. After 20 min incubation at 37 °C, additional 25 mL DMEM + 2% FCS were added, and cells were incubated for 48 h. Thereafter, supernatant was transferred into a 50 mL tube and centrifuged for 20 min at 3220× *g* at 4 °C to remove cell debris. The clarified supernatant was then aliquoted and stored at −80 °C. Before the stock was used, virus titer was determined by focus-forming assay and diluted to desired concentrations and verified by focus-forming assay.

### 2.4. Focus-Forming Assay

Infectious viral titers were quantified by focus-forming assay. MC57G cells were prepared at 5 × 10^5^ cells/mL. For titration, 96-well plates were prefilled with 135 µL DMEM + 2% FCS in rows 2–12. Samples (200 µL) were added in duplicate to row 1 and serially diluted by transferring 65 µL across the plate, resulting in a 1:10 dilution for every second row. For infection, 200 µL of the MC57G cell suspension was added to each well of a 24-well plate, and only every second dilution from the 96-well plate (corresponding to the 1:10 steps) was transferred onto the cells. After 4 h, once cells had adhered and virus binding had occurred, cultures were overlaid with 1% methylcellulose (Sigma-Aldrich, 64632) in IMDM (Gibco, 12200069) to restrict viral spread and permit discrete focus formation. After 48 h, cells were fixed with 4% formalin (AppliChem GmbH, Darmstadt, Germany, 141328), permeabilized with 1% Triton X-100 (Sigma-Aldrich, T9284), and blocked with 10% FCS. Viral foci were detected using a rat anti-LCMV NP antibody (Clone VL4, produced in-house), followed by an HRP-conjugated anti-rat secondary antibody (Jackson immunoresearch laboratories, West Grove, PA, USA, 112-035-003). O-Phenylenediamine Dihydrochloride (OPD, Sigma-Aldrich, P8412) substrate was applied to visualize infected foci which were counted and the FFU calculated accordingly.

### 2.5. Subcloning of Virus

To obtain single mutant viruses, subcloning of mixed stocks and passages was performed. Firstly, 5 × 10^4^ BHK-21 cells were seeded in a 24-well plate and infected with different titrations of the virus stock per row. Starting with the titer measured by focus-forming assay, the virus-containing sample was diluted with DMEM + 2% FCS to a final concentration of 50 FFU/mL. This material was used as starting point for 3 dilution steps. A 200 µL volume of respective dilutions were added to the cells in six replicates. Statistically, the lowest row contained < 1 FFU per well. After 72 h, the supernatant was sampled and stored, and the cells were stained for viral NP. A positive staining reveals the initial presence of at least one virus particle. If the staining was only detectable in a few wells of the same dilution, the limiting dilution was successful. The stored supernatant of 3–5 clones was used for sequencing and clones with the desired sequence were used to produce new stocks.

### 2.6. Infectivity Assay

The infectivity assay determines the capacity of a virus to enter the cell, replicate in the cell and produce virus proteins. In this assay, cells (i.e., Neurons, H1975, Gist-T1, MaMel86a, MC57G, and MaMel51 cells directly after harvesting) are incubated with virus (multiplicity of infection of 0.01 to 0.1) at 37 °C for 16 h (in flat bottom 96-well plates in 200 µL of the respective cell culture medium). During this incubation time, the virus is propagating in the cell culture. Virus particles, which are produced within the cell culture, can infect new cells. After the incubation time, virus protein is determined in each cell by staining the cells with an anti-LCMV-NP antibody (Clone VL4) and analysis by flow cytometer. To do so, cells are harvested with Trypsin/EDTA, fixed for 10 min with 2% Formalin and then washed two times with FACS Buffer (PBS, 1% FCS, 5 mM EDTA and 0,1% Sodium azide) with 0,1% Saponin (Sigma-Aldrich, 1003147511) added. After washing, cells are stained with anti-LCMV-NP (VL4, produced in-house) for 30 min. After two additional washes, the primary antibody is detected with a fluorescently labeled anti-rat antibody (Jackson immunoresearch, 112-116-072) by flow cytometry. The percentage of infected cells reflects viral propagation efficiency in the respective cell culture.

### 2.7. Virus Sequencing

Viral RNA was isolated from infected cell supernatants or stocks using QIAamp Viral RNA Mini Kit (Qiagen GmbH, Hilden, Germany, 52904) by standard protocol. cDNA was prepared with SuperScript IV RT (Thermo Fisher Scientific, Waltham, MA, USA, 18090050) following the manufacturer’s instructions and using a primer binding to the 3′ ends of both genomic RNA strands. Before Sanger Sequencing, the single-stranded cDNA was amplified in 5 subsegments. For the PCR setup, 25 µL nuclease-free water, 10 µL 5X iProof HF buffer, 1 µL dNTPs, 10 µL 5M betaine, 0.5 µL iProof High-Fidelity DNA Polymerase (Bio-Rad Laboratories, Hercules, CA, USA) and 2.5 µL of the respective primer pair (10 µM each) were mixed and 1 µL cDNA template was added.

### 2.8. Bone Marrow Isolation

Bone marrow cells were isolated under sterile conditions from tibiae and femurs of mice. Bones were cleaned from surrounding tissue, briefly sterilized in 70% ethanol (Honeywell International Inc., Charlotte, NC, USA, J1630) for 3 min, and transferred to PBS (Pan Biotech, P04-36500). Bone marrow was flushed from the bones using RPMI-1640 medium (PAN Biotech, P04-17525) with stable glutamine and low endotoxin levels to obtain a single-cell suspension. Cells were passed through a 70–100 µm cell strainer, erythrocytes were lysed with EryLyse buffer (Pan Biotech, P10-90100) for 3 min, and the reaction was stopped by adding 10 mL medium prior to centrifugation (300× *g*, 5 min). Cells were then resuspended in the RPMI-1640 medium supplemented with 10% FBS (Gibco, A5256801), 50 µM β-mercaptoethanol (Gibco, 31350010), 1% penicillin-streptomycin-L-glutamine solution (Sigma-Aldrich, G6784), and 1% Glutamine (Gibco, 35050061).

### 2.9. pDC Differentiation

For plasmacytoid dendritic cell (pDC) generation, bone marrow cells were seeded at 2 × 10^7^ cells per 10 cm non-treated Petri dish in 10 mL medium supplemented with 100 ng/mL recombinant FLT3L (R&D Systems, Minneapolis, MN, USA, 427-FL-025/CF). Cells were cultured at 37 °C in a humidified atmosphere with 5% CO_2_. On day 5, half of the culture medium was replaced after centrifugation with fresh medium containing FLT3L. Cells were harvested on day 7 and used for experiments.

### 2.10. BMDC Differentiation

For bone-marrow-derived dendritic cell (BMDC) generation, bone marrow cells were seeded at 2 × 10^6^ cells per 10 cm non-treated Petri dish in 10 mL medium supplemented with 40 ng/mL GM-CSF (PeproTech, Cranbury, NJ, USA, 315-03) and 20 ng/mL IL-4 (PeproTech, 214-14). On day 3, the culture volume was doubled by adding fresh medium containing the same supplements. On day 6, half of the culture supernatant was removed, and cells were pelleted by centrifugation (300× *g*, 5 min) then resuspended in fresh medium supplemented with 20 ng/mL GM-CSF and 10 ng/mL IL-4. Cells were harvested on day 7 and used for experiments.

### 2.11. pDC Infectivity Culture

In vitro generated pDCs were co-cultured with MC57G cells and infected with either LCMV-WE or LCMV-P52 at a multiplicity of infection (MOI) of 0.1 or 1 and incubated at 37 °C in a humidified incubator with 5% CO_2_. After 48 h of co-culture, cells were harvested and analyzed by flow cytometry. pDC infection was determined by intracellular staining for LCMV nucleoprotein (NP) using an anti-LCMV-NP antibody (clone VL4). For analysis, pDCs were identified as CD3^−^ (BD Biosciences, 551163), CD19^−^ (BD Biosciences, 551001), CD11c^+^ (Thermo Fisher Scientific, 25-0114-82), CD11b^−^ (Thermo Fisher Scientific, 47-0112-82), B220^+^ (Thermo Fisher Scientific, 11-0452-82), mPDCA-1^+^ (BioLegend, 127009), and Siglec-H^+^ (BioLegend, 129611) cells.

### 2.12. Mice

All animal experiments were performed according to the German laws for animal protection and were authorized by the Landesamt für Natur-, Umwelt- und Klima NRW (LANUK) Nordrhein-Westfalen. All mice were housed in individually ventilated cages, checked daily, and sacrificed with corresponding termination criteria or due to experiment settings. Termination criteria were body weight, general condition, spontaneous behavior, clinical findings, and tumor size. C57BL/6 (strain: 632, Charles River Laboratories, Wilmington, MA, USA) mice are a wild-type inbred strain commonly used for immunological studies. Unless otherwise stated, all experiments were performed using female C57BL/6 mice aged 6–8 weeks.

### 2.13. Intracellular Cytokine Staining

Splenocytes were resuspended in DMEM with 2% FCS containing the indicated peptide at 1 µg/mL. After 1 h at 37 °C, Brefeldin A (Sigma-Aldrich, B5936) was added to a final concentration of 10 µg/mL to each well, and cells were incubated overnight. Cells were pelleted and the surface antigens CD4 and CD8 were stained with αCD4-PE and αCD8-PE-Cy7 antibodies (eBiosciences, San Diego, CA, USA). After fixation with 2% Formalin, cells were permeabilized and intracellularly stained for IFN-γ (eBioscience, 17-7311-82) and TNF-α (eBioscience, 48-7321-82) and measured with a LSRFortessa flow cytometer (BD Biosciences, San Jose, CA, USA).

### 2.14. Tetramer Staining of Blood

For the tetramer staining, 20 µL of whole blood was stained with APC-labeled GP33-TET for 15 min at 37 °C. After tetramer binding, samples were stained for CD8 with a PE-Cy7-labeled antibody (eBiosciences). Red blood cells were lysed with 1 mL BD FACS Lysing Solution. The samples were recorded with a LSRFortessa.

### 2.15. Immunofluorescence Imaging

Snap-frozen spleen tissues embedded in TissueTek O.C.T. Compound were placed in a cryotome (Leica CM3050 S cryostat, Leica Biosystems, Nussloch, Germany) and cut into 6–8 µm thick tissue sections. After drying (3 h, RT) samples were fixed in acetone for 10 min. Unspecific binding sites were blocked with PBS containing 1% FCS. For immunofluorescence staining, monoclonal antibodies directed against LCMV nucleoprotein (LCMV-NP; clone VL4, generated in-house, 1:1), Biotin-SP anti-rat IgG secondary antibody (112-065-167, Jackson ImmunoResearch, 1:150), Streptavidin (405207, BioLegend, 1:75), and CD169 (142419, BioLegend, 1:100) were used. All antibodies were diluted in PBS supplemented with 2% FCS and incubated on the sections for 45 min at room temperature. Between staining steps, slides were washed three times with PBS. LCMV-NP staining was performed using the VL4 primary antibody, followed by the Biotin-SP antibody and the Streptavidin detection. Anti-CD169 staining was performed simultaneously with the remaining fluorophore-conjugated antibodies at the dilutions indicated above. After staining, slides were washed with PBS, mounted using fluorescence mounting medium, coverslipped, and dried protected from light for at least 30 min prior to imaging. Fluorescence images were acquired using a Keyence BZ-9000 imaging system (Keyence, Osaka, Japan). No blinding was applied during image acquisition or qualitative evaluation of the immunofluorescence images.

### 2.16. IFN-α ELISA

The serum levels of IFN-α were measured with an ELISA kit form Thermo Fisher Scientific according to manufacturer’s instructions. Finally, absorbance at 450 nm was measured using a Cytation 1 multimode plate reader (BioTek Instruments, Winooski, VT, USA). A standard curve was constructed, and sample values were calculated based on it.

### 2.17. Measurement of LDH, ALAT and ASAT

To assess the serum levels of Lactate dehydrogenase (LDH), Alanine transaminase (ALAT), and Aspartate transaminase (ASAT), 30 µL of sample was diluted 1:10 in PBS and analyzed by the Central Laboratory of the University Clinic Essen.

### 2.18. Statistical Analysis

Data were analyzed and visualized using GraphPad Prism version 7. The statistical tests used for each dataset are specified in the corresponding figure legends. In most cases, ordinary one-way ANOVA or two-way ANOVA was applied. Data are presented as the mean ± standard error of the mean (SEM). The level of statistical significance was set at *p* < 0.05. Statistical significance is indicated as * *p* < 0.05, ** *p* < 0.01, *** *p* < 0.001; # *p* < 0.0001.

## 3. Results

### 3.1. Passage of LCMV-WE in H1975 Cells Resulted in Mutations I181M and R185W in the GP Protein

**Figure 1:**
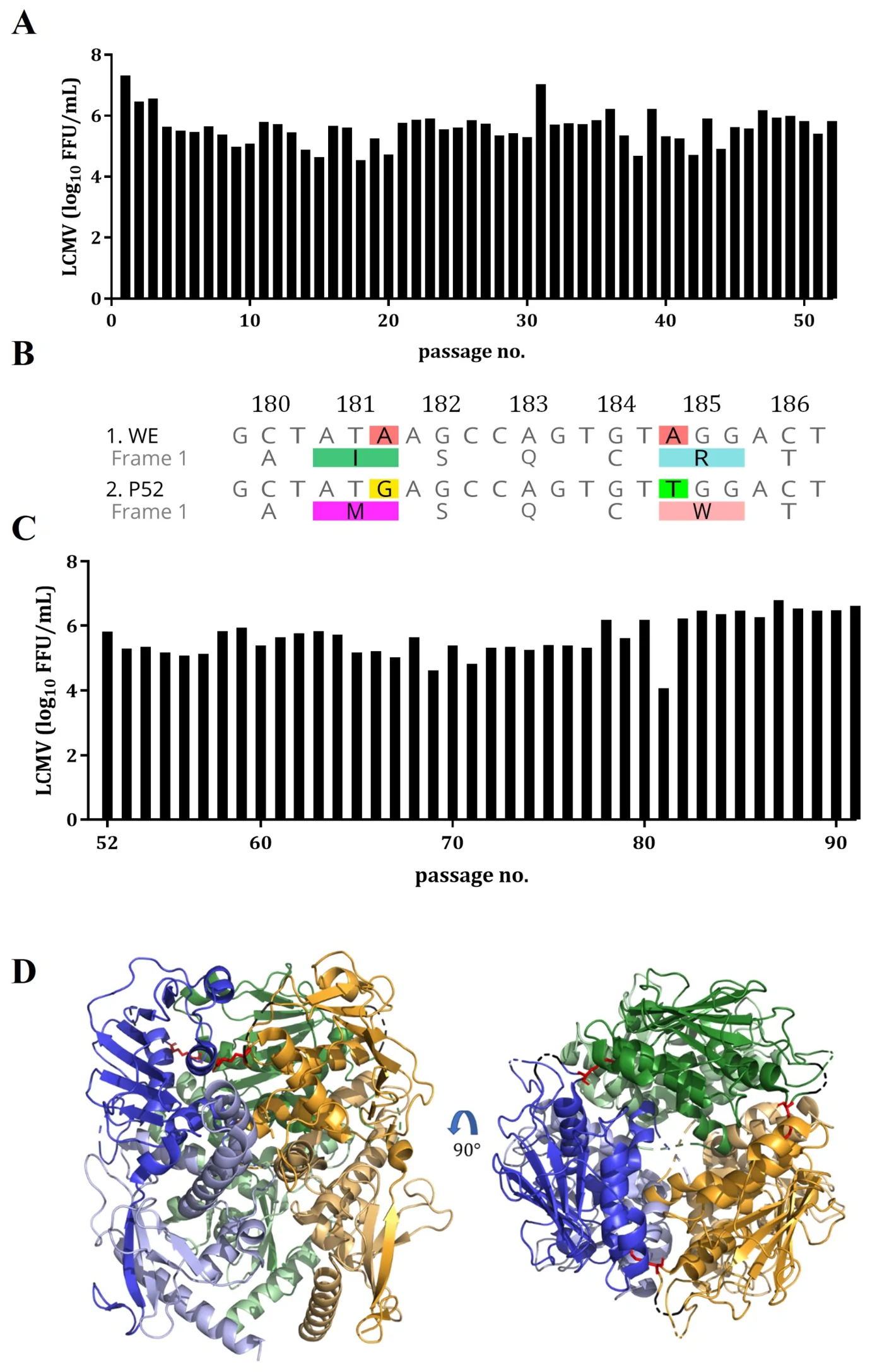
Stable GP mutations I181M and R185W emerge after serial passaging of wild-type strain LCMV-WE in human tumor cells. (**A**) Viral titers at the indicated passage numbers. The titer of supernatants was determined by focus-forming assay. (**B**) Sequence alignment of WE and P52 of the coding sequences for amino acids 180–186 of viral glycoprotein. LCMV mutant P52 harbors two mutations in the viral glycoprotein, I181M and R185W. Amino acid substitutions at positions 181 and 185 are highlighted by colored boxes. Sequence alignment was created using Geneious Prime (version 2026.1). (**C**) Viral titer at the indicated passage numbers. The titer of supernatants was quantified by focus-forming assay. (**D**) Side and top view of estimated LCMV GP trimer. The monomers are colored blue, orange and green, and the GP2 subunit in light colors. The mutated amino acids in position 181 (loop 2, black) and 185 (red) are highlighted. The structure was calculated based on published models of LCMV-GP monomer (5ine) and Lassa-GP trimer (5vk2) using PyMol (version 3.1.8).

To identify naturally acquired mutations arising during adaptation to tumor cells, wild-type LCMV-WE was serially passaged in H1975 human non-small cell lung cancer cells. The viral titer of each passage was determined by focus-forming assay to confirm viral presence and estimate possible impacts on the replication capacity of the virus (Figure 1A). During passaging, viral titers in the supernatant remained relatively stable between 10^5^ and 10^6^ FFU/mL, with the exception of passage 1 and passage 31. The virus population obtained after 52 passages was named “P52” and sequencing was performed. LCMV-P52 (also named WE-G-181) harbors two point mutations in the GP gene: the first mutation replaced the ATA codon by an ATG, resulting in an amino acid exchange at position 181 from isoleucine (I) to methionine (M); the second mutation at position 185 resulted in an AGG to TGG exchange, coding for a tryptophan (W) instead of an arginine (R) (Figure 1B).

To assess the stability of the mutations occurring in P52, serial passaging was continued for an additional 39 passages, resulting in the generation of virus strain “P91”. The viral titers of passages 53 to 91 were determined, and the supernatants almost consistently showed stable titers (Figure 1C). Sequencing of the virus obtained in P91 revealed 100% identity in the S-segment with the P52 virus. Interestingly, the two mutations are located between the Beta 6 (β6) and the Alpha 3 (α3) domains, and are therefore located at a site of LCMV-GP trimer formation and receptor engagement (Figure 1D) [15]. In conclusion, extended passaging resulted in two stable mutations, I181M and R185W, within the LCMV-GP protein.

### 3.2. Both Mutations Were Introduced During Passage 29 as Two Separate S-Segments

**Figure 2:**
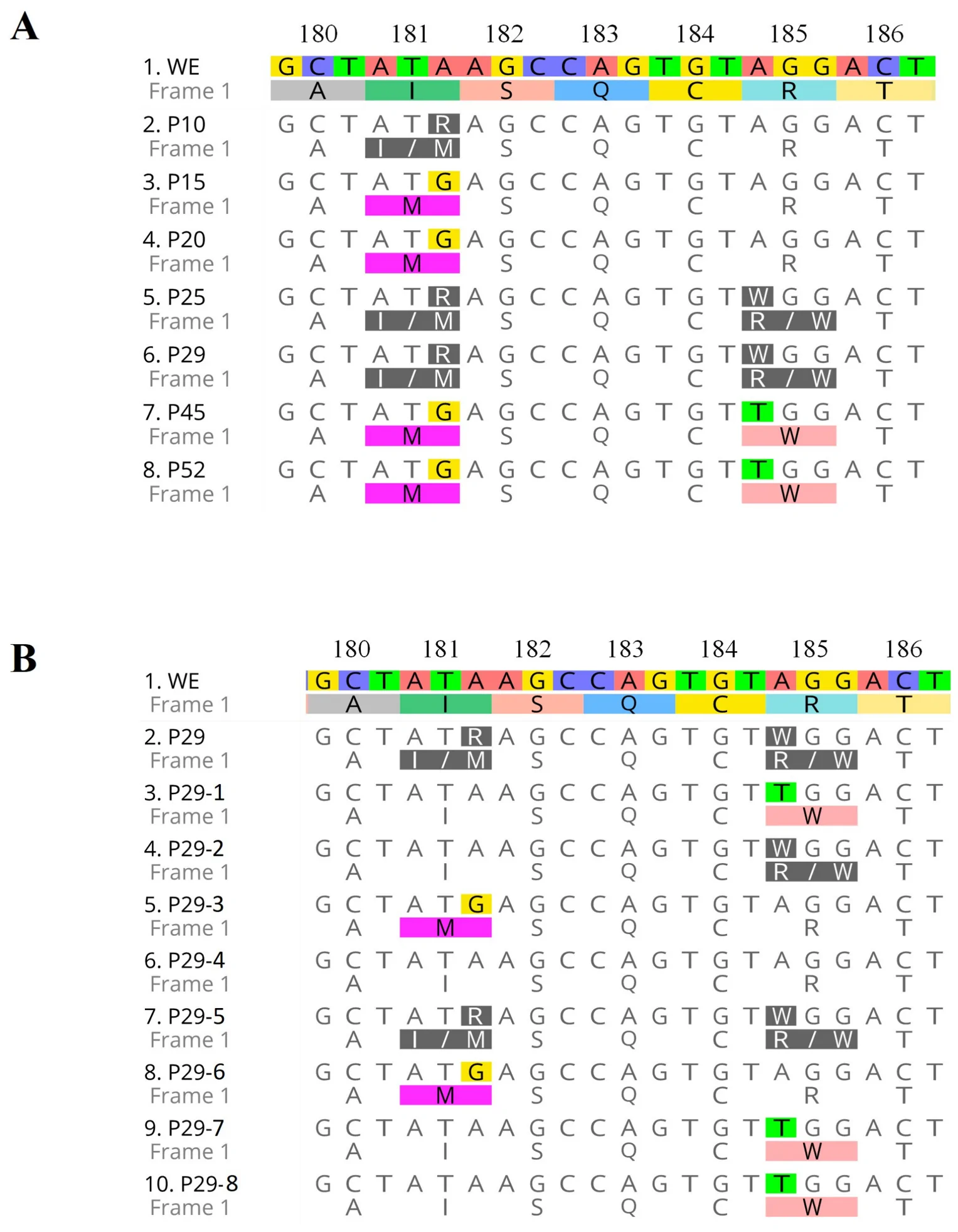
Mutations I181M and R185W arise independently and recombine during serial passaging. Different colored boxes highlight distinct amino acid substitutions. (**A**) Sequence alignment of passages starting at P10 or P29. Virus passages were sequenced, and history of mutations became visible compared to LCMV-WE. (**B**) Limiting dilution of passage 29 and sequencing of viral subclones identified separate clones carrying either the I181M or the R185W mutation, compared with LCMV-WE and the mixed viral population of LCMV-P29.

Having identified two stable GP mutations, we next investigated when these mutations emerged during serial passaging and whether they arose independently or together. The I181M mutation was detected as early as passage 10 (Figure 2A), whereas the R185W mutation was first observed in passage 25 (Figure 2A). From these data, it remained unclear whether the R185W mutation developed in a virus already carrying the I181M mutation or independently in the wild-type virus. The mutations I181M and R185W dominated during passages 25 to 29; however, wild-type sequences were still detectable. To further investigate the origin of the R185W mutation, viruses from passage 29 were subcloned. After limiting dilution of P29, eight subclones were isolated and sequenced. Some subclones contained both mutations, which might be due to insufficient dilution (Figure 2B). However, two separate virus subclones with either the I181M or the R185W mutation were identified (Figure 2B). These findings indicate that both mutations occurred separately in the wild-type virus. Upon further passaging, a dominating virus occurred that harbored both mutations I181M and R185W, suggesting that these amino acid positions represent potential preferential sites for mutations in the GP protein. For other RNA viruses, a template switch of the RNA polymerase is known, which explains the fast recombination ability of the viruses [16,17,18]. Therefore, the LCMV RNA polymerase may switch between two different S-segments, which could explain why the two mutations were found to be combined after only a few passages.

### 3.3. In Vitro Replication and Infectivity of Mutant Viruses

**Figure 3:**
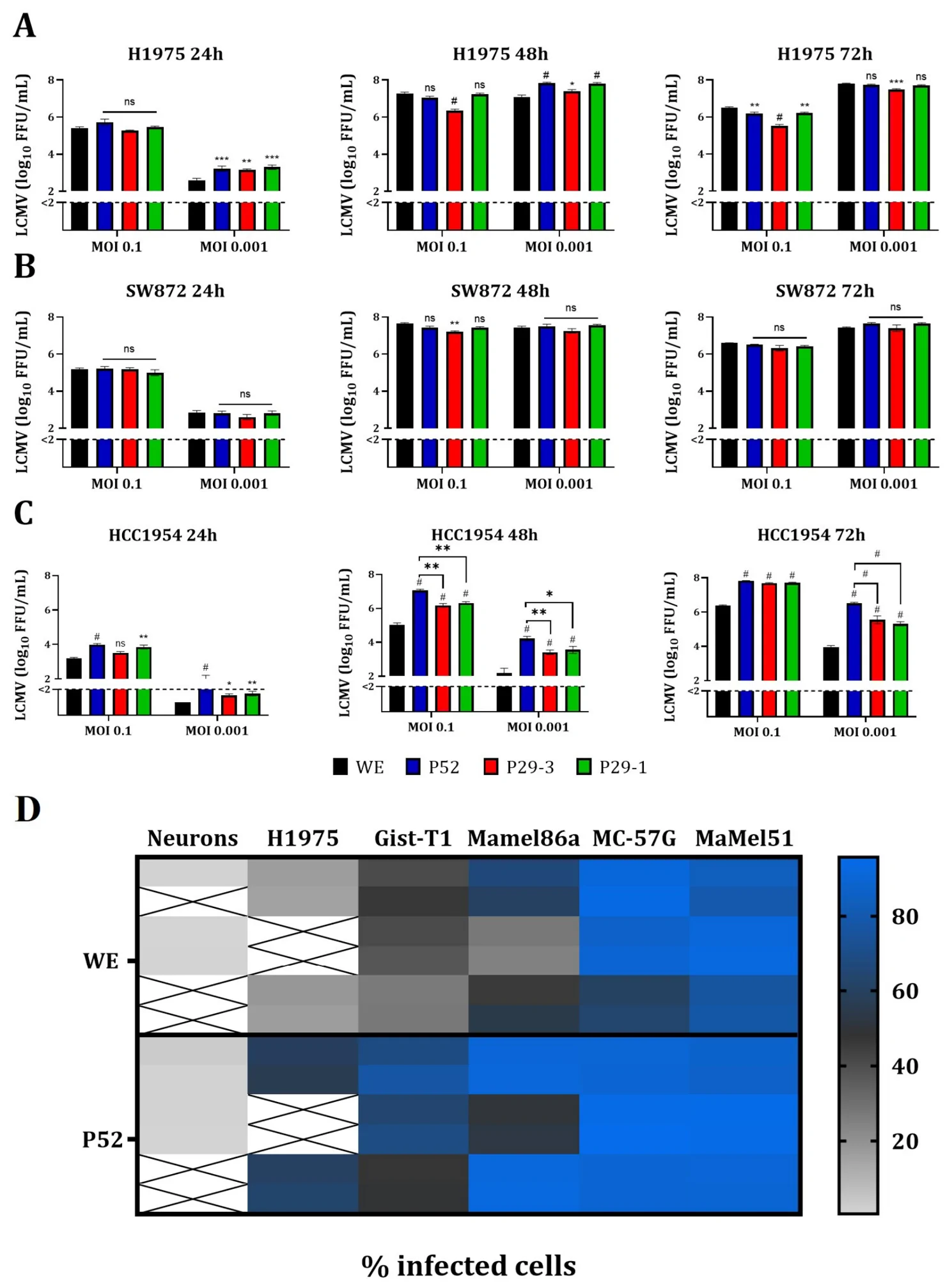
GP mutations enhance LCMV replication and infectivity in human tumor cells. (**A**–**C**) Replication and infection capability in vitro. Human cancer cell lines H1975 (**A**), SW872 (**B**) and HCC1954 (**C**) were infected with different MOIs (0.1 and 0.001) and infectious titers in supernatants were determined by focus-forming assay (*n* = 3–6, duplicates from 3 independent experiments). In (**A**–**C**), the horizontal dashed line indicates the assay detection limit. Data are presented as the mean ± SEM. Statistical analysis was done by two-way ANOVA with symbols indicating *p*-value in comparison to LCMV-WE; ns: not significant, *: *p* < 0.05, **: *p* < 0.01, ***: *p* < 0.001, #: *p* < 0.0001. (**D**) Infectivity (MOI 0.1, 16 h p.i.) of different human healthy (Neurons) and tumor cells (H1975, Gist-T1, MaMel86a, MC57G, MaMel51) was measured by flow cytometry for LCMV-WE and P52 (*n* = 3–6, singlets or duplicates from 3 independent experiments).

To determine the functional consequences of the GP mutations, we next assessed their effects on viral replication and infectivity in vitro. Three human cancer cell lines were infected with different multiplicities of infection (MOIs), and viral titers in the supernatants were analyzed by focus-forming assay at different timepoints. All mutant viruses, P52 and the single mutants P29-3 (mutation at position 181) and P29-1 (mutation at position 185), showed significant differences in their replication compared with the wild-type strain LCMV-WE. In H1975 cells, the cell line used for viral adaptation, P52 showed slightly increased titers after 24 h at an MOI of 0.1, and significantly higher titers at an MOI of 0.001 after 24 and 48 h (Figure 3A). After 72 h, replication of LCMV-WE reached titers similar or higher than those of the mutant viruses (Figure 3A). At early timepoints, both single mutants were equivalent to the double-mutated virus, whereas after 48 h, P29-1 displayed a replication pattern resembling the P52 phenotype in H1975 cells at an MOI of 0.001. However, in SW872 (liposarcoma) tumor cells, neither the single-mutant nor the double-mutant viruses showed any significant differences in replication at any measured timepoint or MOI compared to LCMV-WE (Figure 3B). In HCC1954 (breast cancer) cells, all three mutant viruses demonstrated increased propagation compared with the wild-type strain (Figure 3C). These findings suggest that the replication advantage conferred by the I181M and R185W mutations is cell-type dependent, indicating that host cell-specific factors influence the biological consequences of these mutations. The replication advantage of the double-mutant virus was evident in HCC1954 cells at 48 h at both MOIs (0.1 and 0.001) and remained significant at 72 h at an MOI of 0.001. These results demonstrate that the impact of the mutations on viral propagation varies across tumor cell lines, with the most pronounced advantage of the double-mutant virus observed in HCC1954 cells.

Next, the effect of the two mutations on infection efficiency in tumor cells was assessed. A flow-cytometry-based assay was established to determine cellular virus infection via measuring the LCMV nucleoprotein (NP) at 16 h post-infection, comparing P52 with wild-type strain LCMV-WE (Figure 3D). As expected, P52 showed moderately to markedly increased single cell infection across all cancer cell lines tested compared to LCMV-WE, without increasing infection in primary neuron cultures, indicating that the enhanced infectivity remains restricted to tumor cells (Figure 3D). In conclusion, P52 exhibits enhanced early infectivity across multiple human tumor cell lines, which may contribute to viral propagation in a cell type-dependent manner. These findings indicate that although P52 exhibits enhanced early infectivity across multiple tumor cell lines, the overall propagation phenotype is additionally determined by host cell-specific factors.

### 3.4. In Vivo Replication of LCMV-P52

**Figure 4:**
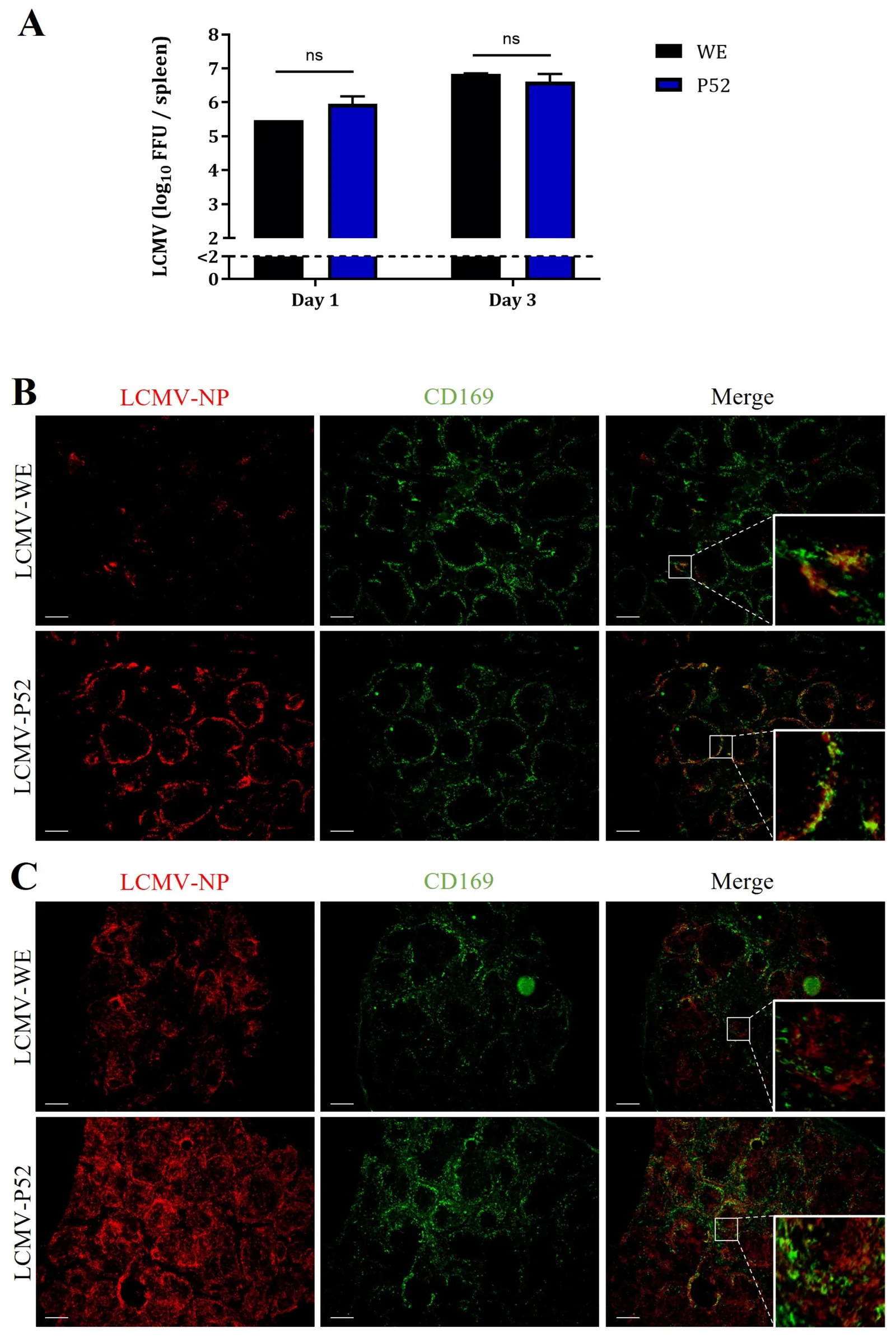
LCMV-P52 shows rapid splenic spread and infection of CD169^+^ macrophages. (**A**) C57BL/6 mice intravenously infected with 2 × 10^4^ FFU of indicated virus were sacrificed on day 1 or 3, and virus titer in spleen was determined by focus-forming assay (*n* = 3, mice/group). The horizontal dashed line indicates the assay detection limit. Data are presented as the mean ± SEM. Statistical analysis was done by two-way ANOVA with symbols indicating *p*-value; ns: not significant. (**B**,**C**) Immunofluorescent staining of spleen sections. C57BL/6 mice were infected with 2 × 10^4^ FFU of LCMV-WE or LCMV-P52 i.v. and spleens of infected mice were stained for LCMV-NP (red) and CD169 (green) on day 1 (**B**) and day 3 (**C**) after infection. Scale bars, 200 µm (*n* = 3, mice/group).

Having demonstrated enhanced infectivity in vitro, we next investigated whether LCMV-P52 also exhibits altered replication and tissue distribution in vivo. C57BL/6 mice were infected intravenously with 2 × 10^4^ FFU of LCMV-WE or LCMV-P52, and virus distribution in spleen was analyzed on days 1 and 3 post-infection. Viral titers in spleen homogenates, determined by focus-forming assay, showed no significant differences between the two virus strains (Figure 4A). To gain more detailed insight into the splenic distribution of the virus, immunofluorescent staining of tissue sections was performed. In line with published data [19], the infection with 2 × 10^4^ FFU of LCMV-WE resulted in only few infected cells, mainly CD169^+^ marginal zone (MZ) macrophages after one day of infection (Figure 4B). In contrast to LCMV-WE, LCMV-P52 showed fast distribution within CD169^+^ macrophages (Figure 4B). Three days after infection, the MZ architecture was partially disrupted, and the viral spread into both the red and white pulp was evident (Figure 4C). While infection of the red pulp was rarely visible following LCMV-WE infection, LCMV NP protein expression could be observed in almost the entire splenic tissue in LCMV-P52-infected mice (Figure 4C). Together, these data show that LCMV-P52 infects large numbers of CD169^+^ macrophages. Since early viral replication in CD169^+^ macrophage strongly depends on the expression of the IFN-I inhibitor USP18 as well as IFN-I induction by pDCs [20], we hypothesized that differences in pDC activation might be responsible for the observed phenotype.

### 3.5. The Mutations I181M and R185W Induce Decreased Systemic IFN-I Levels

**Figure 5:**
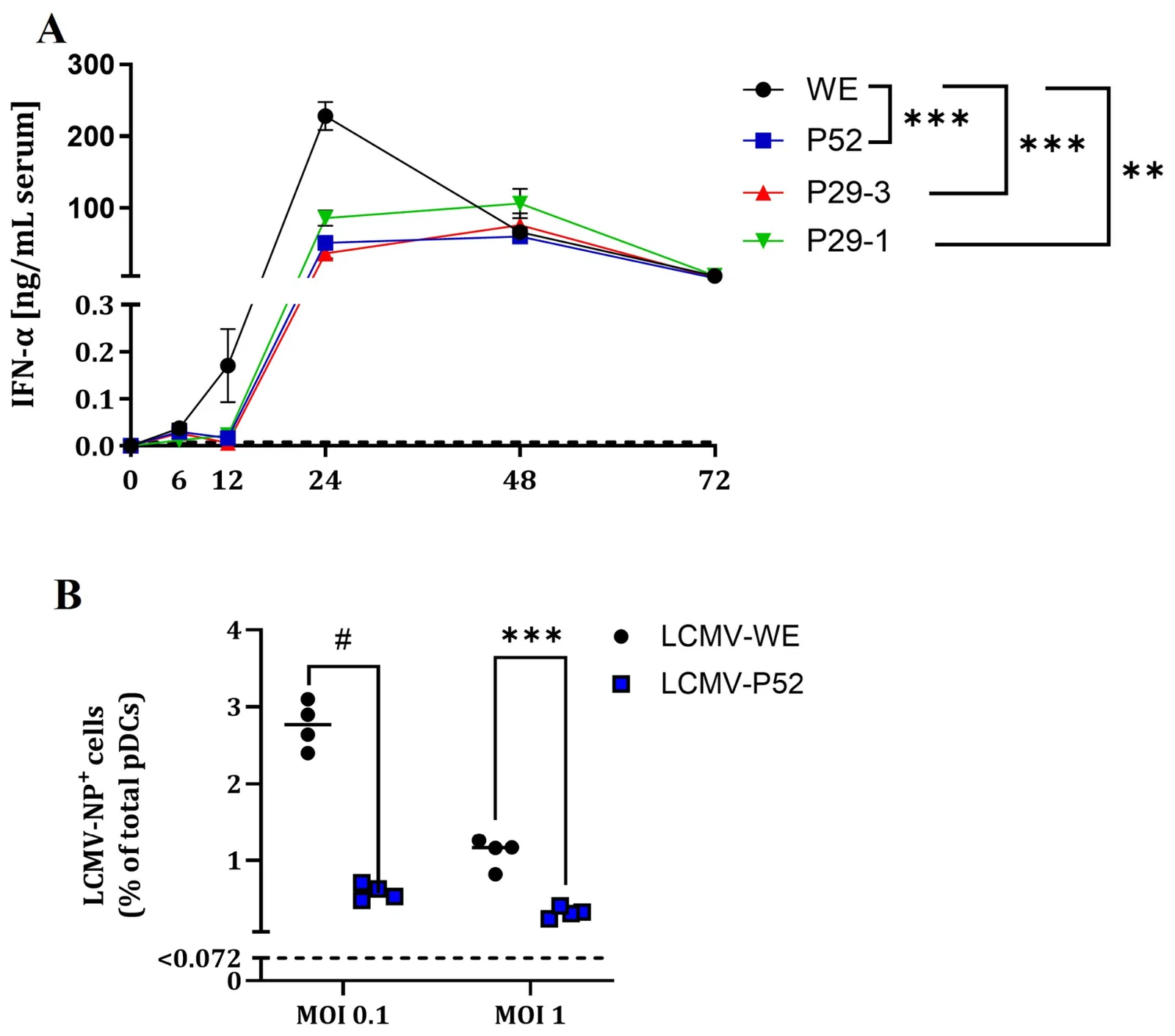
GP mutations limit IFN-I induction through reduced infection of pDCs. (**A**) Serum IFN-α levels at indicated timepoints. Mice were bled 6, 12, 24, 48 and 72 h post-infection (h.p.i.), and IFN-α levels were measured by ELISA. For 6 and 12 h, *n* = 3, mice/group; for all other timepoints, *n* = 6, mice/group. Statistical analysis is shown for 24 h.p.i. (**B**) Bone-marrow-derived pDCs were co-cultured with MC57G cells and then infected with either LCMV-WE (MOI 0.1 and 1) or LCMV-P52 (MOI 0.1 and 1). Expression of LCMV-NP was determined after 48 h. One of two independent experiments with similar results is shown. The horizontal dashed line indicates the assay detection limit. Data are presented as the mean ± SEM. Statistical analysis was done by two-way ANOVA with symbols indicating *p*-value; **: *p* < 0.01, ***: *p* < 0.001, #: *p* < 0.0001.

Because early viral replication in APCs is tightly regulated by IFN-I, we next investigated whether the GP mutations alter the early innate immune response in vivo. Recent studies have demonstrated that a lack of IFN-I signaling in APCs early after infection is essential for the generation of effective antiviral immunity [3,4,5]. To investigate whether a limited IFN-I induction correlates with the accelerated propagation of LCMV-P52 within the marginal zone, C57BL/6 mice were infected intravenously with 2 × 10^4^ FFU of each virus strain, including the single mutants, and serum IFN-α levels were quantified by ELISA. All mutant viruses showed reduced IFN-α levels after 12 and 24 h compared to the wild-type LCMV-WE strain, whereas levels became comparable after 48 and 72 h, when IFN-α concentrations in LCMV-WE-infected mice decreased (Figure 5A). These results indicate that the GP mutations are associated with reduced early IFN-α induction. Since pDCs are the first cells to produce IFN-I during viral infection, we hypothesized that uptake and/or infection of pDCs is limited during infection with LCMV-P52. To determine whether reduced pDC infection is associated with the diminished IFN-I response, we generated pDCs in vitro and co-cultured them with MC57G cells infected with either LCMV-WE or LCMV-P52, thereby mimicking physiological exposure of pDCs to infect target cells. LCMV-NP expression in pDCs was analyzed after 48 h. As expected, significant amounts of LCMV-NP were detected in pDCs co-cultured with LCMV-WE-infected cells (Figure 5B). In contrast, pDCs showed only limited LCMV-NP expression when infected with LCMV-P52 (Figure 5B). Next, we examined whether bone-marrow-derived DCs (BMDCs) also showed less induction of IFN-I after infection with LCMV-P52. Indeed, both IFN-α and IFN-β levels in the supernatant were strongly reduced after infection with LCMV-P52 (Supplementary Figure S1). Collectively, these data demonstrate reduced infection of pDCs by LCMV-P52 and reveal an association between this phenotype and lower systemic IFN-I levels.

### 3.6. The Mutations I181M and R185W Induce Accelerated Antiviral CD8^+^ T Cell Responses

**Figure 6:**
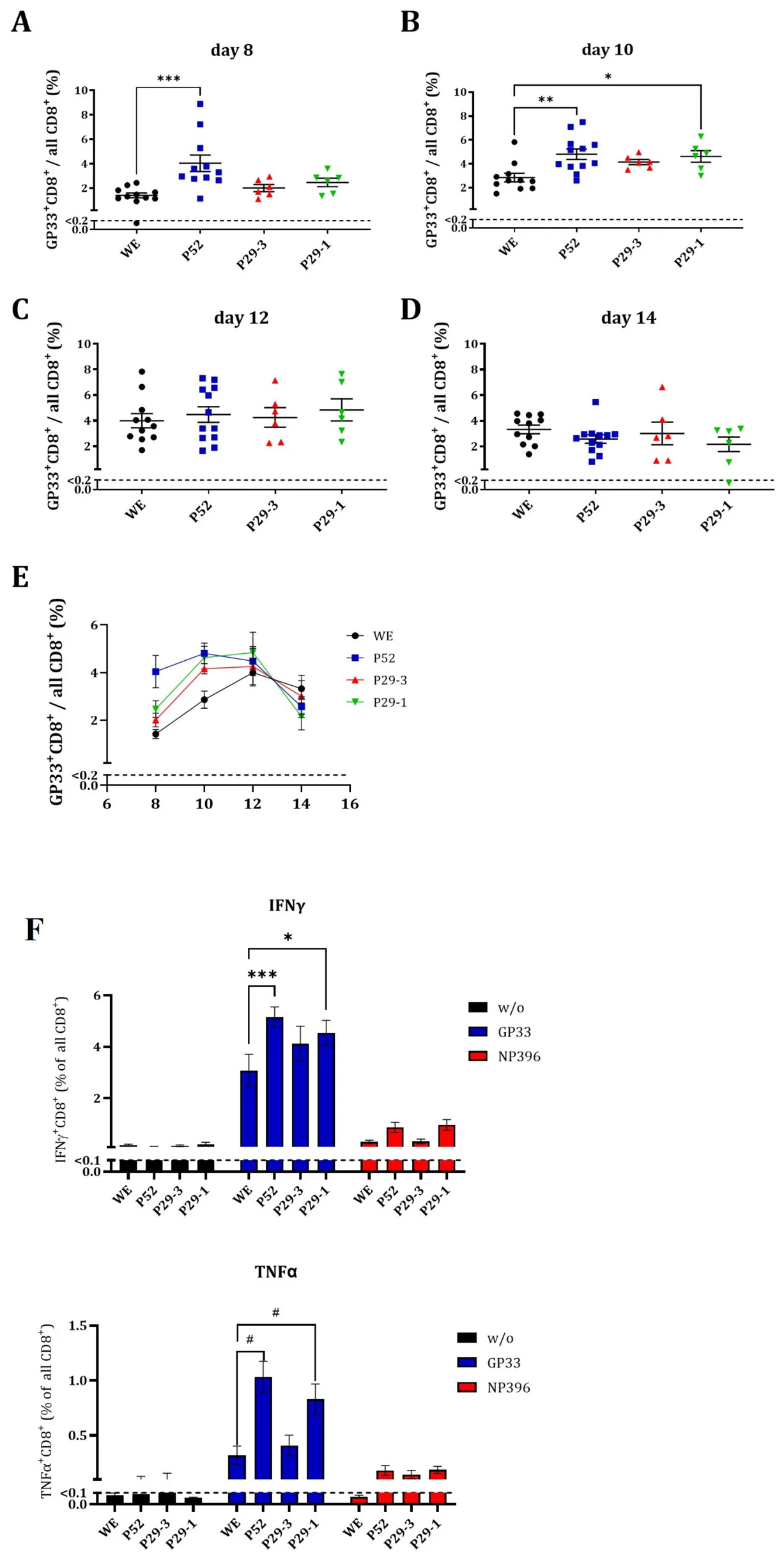
Mutations I181M and R185W accelerate virus-specific CD8^+^ T cell responses. (**A**–**D**) Blood samples from C57BL/6 mice infected with 2 × 10^4^ FFU of indicated virus were assessed for GP33-specific T cells on day 8, 10, 12, and 14 d.p.i. (*n* = 6–12 mice/group). (**E**) Above data is depicted over time. (**F**) Restimulation of splenocytes on day 30. Splenocytes were restimulated overnight with the viral peptides GP33, NP396 or medium only. After 1 h, BFA was added to inhibit secretion of cytokines (*n* = 6, 2 independent experiments with each 3 mice/group). The horizontal dashed line indicates the assay detection limit. Data are presented as the mean ± SEM. Statistical analysis was done by ordinary one-way ANOVA with symbols indicating *p*-value in comparison to WE. *: *p* < 0.05, **: *p* < 0.01, ***: *p* < 0.001, #: *p* < 0.0001.

Given the reduced IFN-I response induced by LCMV-P52, we next investigated whether these changes influence the magnitude and function of antiviral CD8^+^ T cell responses. We determined the antiviral CD8^+^ T cell response by quantifying the frequency of CD8^+^ T cells specific for the viral epitope representing amino acids 33–41 of the viral glycoprotein (GP33). LCMV-P52 induced a faster and stronger antiviral CD8^+^ T cell response compared to LCMV-WE, while P29-3 showed no difference and P29-1 induced only a slight increase in GP33-specific CD8^+^ T cells on day 10 post-infection (Figure 6A–E). To measure the function of LCMV-reactive T cells, splenocytes were isolated on day 30 post-infection and restimulated ex vivo with viral peptides. The intracellular cytokine staining (ICS) showed that infection with LCMV-P52 induces higher frequencies of virus-specific CD8^+^ T cells producing IFN-γ as well as TNF-α in response to GP33 peptide compared to LCMV-WE (Figure 6F). P29-3 did not differ significantly from WE, whereas P29-1 elicited increased frequencies of GP33-specific CD8^+^ T cells producing IFN-γ upon viral peptide stimulation. This effect was also reflected in TNF-α-secreting T cells, further supporting the stronger T cell induction by P52 (Figure 6F). Consistent with these findings, NP396-specific CD8^+^ T cells followed the same pattern, showing non-significant but consistent increase in P52 and P29-1 (Figure 6F). Together, these data indicate that the accelerated propagation of LCMV-P52 in CD169^+^ macrophages induces higher levels of virus-specific CD8^+^ T cells.

### 3.7. The Mutations I181M and R185W Induce Lower Levels of Immunopathology

**Figure 7:**
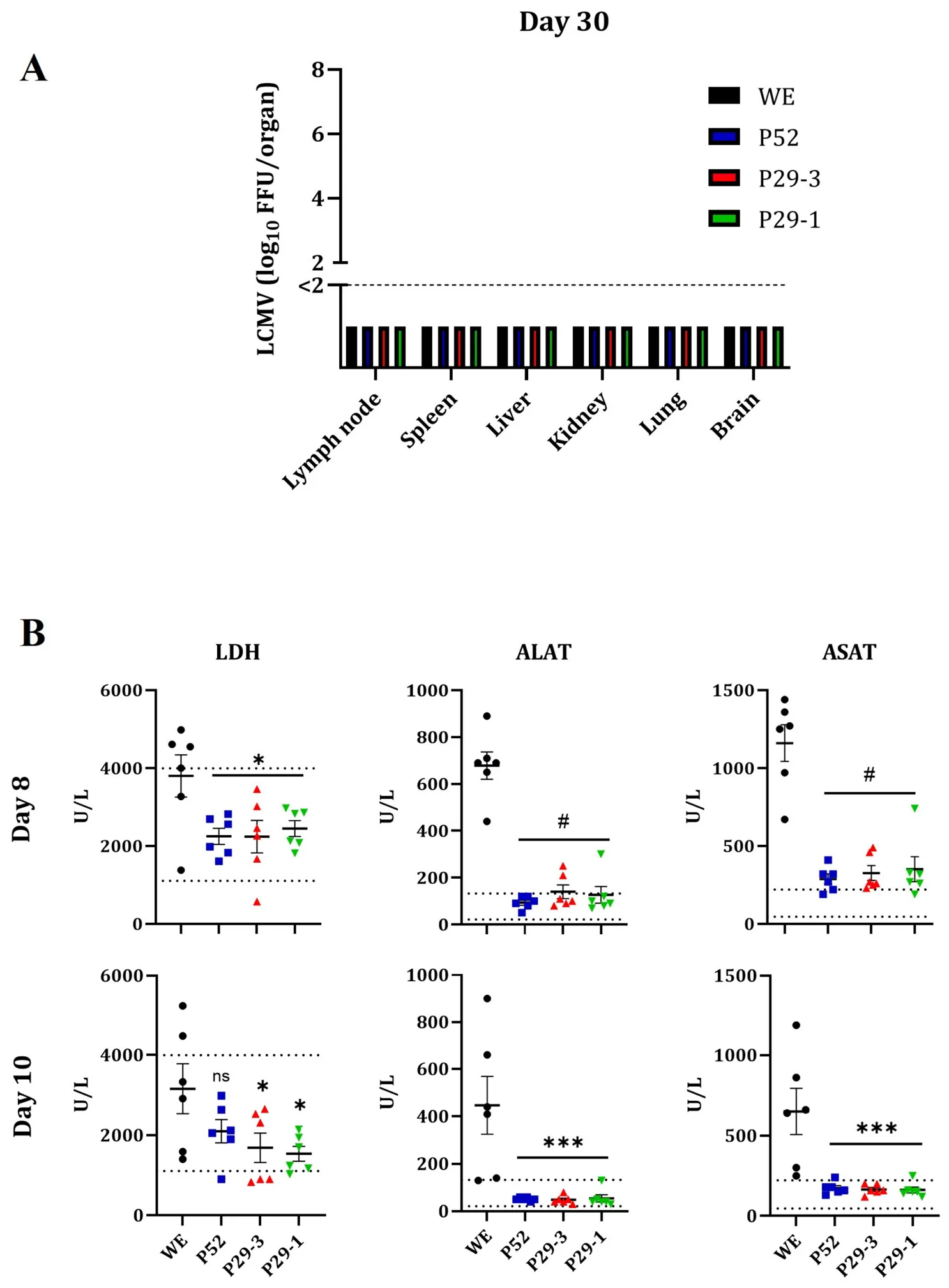
Mutant LCMV strains cause no liver immunopathology while maintaining viral control. (**A**) C57BL/6 mice infected with 2 × 10^4^ FFU of indicated virus were sacrificed on day 30 and organ (lymph node, spleen, liver, kidney, lung, and brain) titers were determined by focus-forming assay (*n* = 6 mice/group). In (**A**), the horizontal dashed line indicates the assay detection limit. (**B**) Activity of hepatic enzymes in serum of infected animals. To assess potential liver damage, serum levels of LDH, ALAT and ASAT were determined on day 8 and 10 of mice infected with 2 × 10^4^ FFU i.v. of indicated virus (*n* = 6 mice/group). In (**B**), the upper and lower horizontal dotted lines indicate the physiological reference range for healthy C57BL/6 mice. Data are presented as the mean ± SEM. Statistical analysis was done by ordinary one-way ANOVA with symbols indicating *p*-value in comparison to WE. ns: not significant, *: *p* < 0.05, ***: *p* < 0.001, #: *p* < 0.0001.

Finally, we investigated whether the altered innate and adaptive immune responses induced by the GP mutations influence viral clearance and the development of immunopathology. Acute infections with LCMV are typically controlled by wild-type mice within 10–20 days. To determine whether the mutated strains are similarly controlled, plaque assays were performed 30 days post-infection. Indeed, LCMV-P52 was controlled in all mice and organs at this timepoint (Figure 7A). The LCMV-WE strain is widely used as a model for virus-induced transient hepatitis in mice [21]. Infection with a high dose of LCMV-WE results in liver infection and consecutive T cell-mediated killing of hepatocytes, leading to increased levels of hepatic enzymes in the serum between days 8 and 10 post-infection. Furthermore, virus strains exhibiting high affinity towards entry receptors can facilitate innate affinity escape and trigger resistance to IFN-I [15,22]. To investigate possible changes in hepatic tropism of the mutant viruses, sera of infected mice were analyzed for lactate dehydrogenase (LDH), alanine aminotransferase (ALAT) and aspartate aminotransferase (ASAT) activity. All three mutant viruses showed clearly reduced levels of LDH, ALAT and ASAT on days 8 and 10 after infection compared with LCMV-WE (Figure 7B). These data indicate that the reduced IFN-I secretion and fast induction of virus-specific CD8^+^ T cells accelerate viral clearance in the liver and correlates with reduced liver pathology in this model of acute LCMV-induced hepatitis.

## 4. Discussion

In this study, we found that passaging of wild-type strain LCMV-WE in human tumor cells results in the induction of mutations in the LCMV-GP proteins. These point mutations enhance viral infection and replication in tumor cells in vitro and affect the antiviral innate and adaptive immune responses in vivo. Most strikingly, the distribution of the virus within the spleen, representing a secondary lymphoid organ, was strongly accelerated by the two mutations.

Even minor changes in the amino acid sequence can impact the overall phenotype of a virus. Especially for LCMV, the two closely related strains Armstrong and its derivate Clone 13 show pronounced differences in their infection characteristics in vivo, while harboring few differences in their amino acid sequence. The Armstrong strain exhibits neurotropism with low affinity to α-dystroglycan (α-DG), whereas Clone 13 has high affinity to α-DG and shows viscerotropism [23]. Specifically, high affinity viruses such as the 155Y mutation exhibit resistance to innate IFN-I resulting into increased viral replication [15,22]. Nevertheless, the GP protein only differs in one amino acid that affects the protein folding, thereby modifying receptor affinity and tissue tropism [24]. Similarly, in our study, the GP mutations I181M and R185W accelerated distribution of the virus in splenic APCs leading to earlier and more efficient CD8^+^ T cell priming.

Previous data demonstrated that amino acids in or around positions 153, 155 and 190 of the GP proteins appear important for receptor binding [15,22]. The cluster formed by these amino acids points outward from the trimeric structure of the LCMV GP complex, facilitating receptor interaction. Furthermore, the amino acid at position 185 is located on the loop face and contributes to receptor binding [15]. Position 185 has also been identified as a neutralizing antibody escape variant, supporting its exposed position on the outer surface of the GP protein [25]. In addition, positions 181 and 182 were also identified in neutralizing antibody studies as escape mutants [26,27]. Therefore, amino acids at positions 181 and 185 both protrude from the GP protein, making them accessible for interactions with cell surface receptors. Alpha-dystroglycan has been demonstrated to represent one receptor for LCMV, although additional cell surface proteins or glycans have been suggested to be involved in viral uptake [28,29,30]. Nevertheless, no study has investigated a potential role for position 181 in receptor binding or infection. It is therefore possible that this and/or the 185 position in the LCMV GP protein either participate in LCMV binding to α-DG or in interactions with an alternative receptor or with a protein involved in cellular entry such as CD164 [2,31]. The cell line-dependent effects observed in this study suggest that the biological consequences of the I181M and R185W mutations depend on intrinsic properties of the target cells rather than representing a universal increase in viral fitness. Potential contributing factors include differences in the expression of viral entry factors, glycosylation patterns affecting receptor accessibility, and intrinsic antiviral signaling of IFN responsiveness. However, these mechanisms were not directly investigated in the present study and therefore remain speculative.

As a surrogate marker for early immune activation during viral infections, type I interferon levels in the serum were determined. Interestingly, LCMV-WE induced significantly higher IFN-I levels after 24 h that equalized after 48 h with LCMV-P52. There are two main pathways responsible for IFN-I induction: either by intracellular RNA sensors like RIG-I and MDA-5, which signal through the mitochondrial antiviral signaling protein (MAVS) as the shared signal transducer, or via membrane bound toll-like receptors (TLRs) that signal via the adaptors myeloid differentiation primary response 88 (MyD88) or TIR domain-containing adaptor-inducing interferon-β (TRIF) in the case of TLR3. As the serum IFN-I levels on day 2 have been reported to depend on MAVS, intracellular RNA sensors seem to be important at that timepoint [32]. In contrast, previous studies reported that MAVS^−/−^ mice exhibit IFN-I responses comparable to WT mice during the first day of LCMV infection [33]. These observations suggest that the early IFN-I response may predominantly involve MAVS-independent pathways, potentially including TLR-mediated sensing, although the relative contribution of these pathways was not directly investigated in the present study.

For LCMV-P52, the viral RNA sequences that can activate innate immune sensors only differ by two nucleotides from the wild-type strain, and its viral NP protein, known to inhibit cellular IFN-I induction [33,34], is identical to LCMV-WE. Therefore, the reduced IFN-I induction observed for LCMV-P52 is more likely associated with the GP mutations with altered viral RNA sensing or NP-mediated immune evasion. The present study demonstrates that the GP mutations I181M and R185W are associated with reduced infection of pDCs and diminished early systemic IFN-I responses. However, the precise molecular basis of this phenotype remains unresolved. One possibility is that the mutations alter interactions with cellular entry receptors such as α-DG or CD164, thereby modifying viral tropism. Alternatively, the mutations may influence post-binding entry events, cell–cell interactions, or early viral dissemination without fundamentally changing receptor usage. Because receptor usage and entry mechanisms were not directly investigated in the present study, these possibilities remain speculative and warrant further investigation. Indeed, pDCs appear to be less efficiently infected by LCMV-P52, which is associated with reduced IFN-I production. Furthermore, TLRs that are known to recognize LCMV mainly detect the viral RNA [35], while TLR2 is the only one able to detect proteins of some viruses, and TLR2^−/−^ mice showed reduced IFN-I bioactivity [7]. Therefore, reduced infection of pDCs likely represents an important contributor to the lower IFN-I induction observed for LCMV-P52, while altered stability or innate immune recognition of the viral GP may further contribute to this phenotype.

We observed a stronger CD8^+^ T cell response following infection with the mutant viruses. This finding is consistent with previous reports suggesting that reduced IFN-I signaling can limit CD8^+^ T cell exhaustion during chronic viral infection [11]. However, as exhaustion markers were not assessed in the present study, this represents a possible explanation rather than a demonstrated mechanism. Here, the ambivalent role of early DC infection becomes visible. DCs usually induce a potent innate immune activation; however, this is accompanied by early DC destruction, which can limit the adaptive immune response [10,11]. In contrast, CD169^+^ macrophages play a key role in priming CD8^+^ T cells [36]. Together with the possibility of reduced CD8^+^ T cell exhaustion, LCMV-P52 showed increased infection of CD169^+^ macrophages in the splenic marginal zone, which may compensate for the lower IFN-I levels and thereby contribute to the enhanced antiviral T cell response.

LCMV-WE is used as a model of virus-induced hepatitis. The destruction of hepatocytes is not induced by the virus itself but by T cell-mediated killing of infected hepatocytes [10,37]. Although LCMV-P52 elicits stronger T cell activation, infected mice displayed significantly decreased serum levels of hepatic enzymes, indicating reduced liver pathology in this acute model of LCMV-induced hepatitis. This phenotype may be associated with the reduced early IFN-I induction observed after infection, which has previously been linked to reduced immunopathology. However, the present study does not distinguish whether the reduced liver pathology primarily reflects altered liver tropism, accelerated viral clearance, or a combination of both mechanisms. Indeed, for hemorrhagic *Mammarenaviruses* such as Lassa virus, it has been demonstrated that the innate immune activation strongly determines arenavirus pathogenesis [38]. Therefore, our findings support the conclusion that the GP mutations are associated with reduced liver pathology in this acute LCMV-induced hepatitis model, while further studies will be required to determine whether these observations extend to other disease settings.

The present study provides mechanistic insight into how the GP mutations I181M and R185W influence viral tropism, innate immune activation, and antiviral T cell responses. These findings complement our previous work demonstrating the therapeutic efficacy and favorable preclinical safety profile of an optimized arenavirus carrying these GP mutations [2], by providing a mechanistic framework that may help explain the improved biological properties observed in this study. While these findings may contribute to the rational development of arenavirus-based therapeutic approaches, the current work is limited to preclinical experimental models. Therefore, any translational implications remain speculative and require further validation.

In conclusion, this work demonstrates that minimal amino acid substitutions in the LCMV GP protein can coordinate changes in viral tropism, innate immune sensing, and the magnitude of adaptive immunity. These findings provide insight into the enhanced anti-tumor properties of LCMV-P52 and highlight GP engineering as a potential strategy to optimize arenaviruses for therapeutic applications, including cancer immunotherapy.

## Data Availability

The data presented in this study are available on request from the principal investigator (PI) Karl S. Lang. The data are not publicly available due to restrictions, e.g., privacy or ethical issues.

## Supplementary Materials

The following supporting information can be downloaded at https://www.mdpi.com/article/10.3390/v18091002/s1. Supplementary Figure S1: LCMV-P52 induces reduced IFN-α and IFN-β secretion by BMDCs.
