# Supplementary material for: Mutations at Positions 181 and 185 Within the LCMV GP Protein Increase Tumor Cell Infectivity, Limit Induction of Type I Interferon, and Accelerate T Cell Activation
Source: Viruses. 2026 Sep 11;18(9):1002. doi: 10.3390/v18091002 (PMC13611988; doi:10.3390/v18091002)
Supplement: Supplementary file 1 [file viruses-18-01002-s001.zip › viruses-4404852-supplementary.pdf]

Supplementary Figure S1

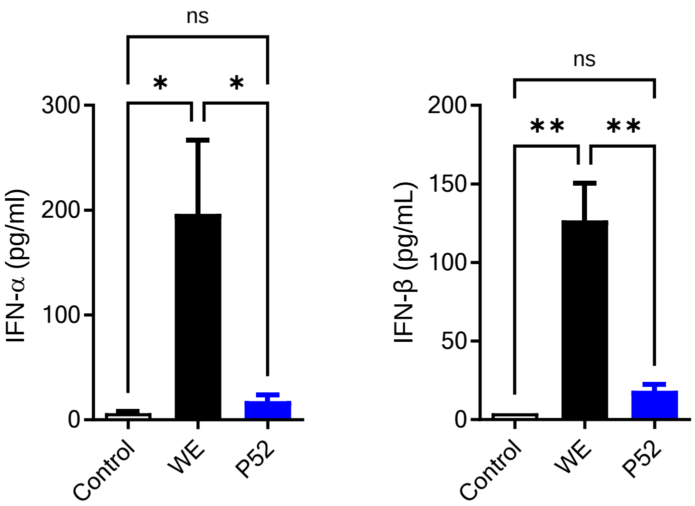

Supplementary Figure S1: LCMV-P52 induces reduced IFN-α and IFN-β secretion by BMDCs

BMDCs were infected with LCMV-WE or LCMV-P52 and IFN-α and IFN-β levels in the supernatant were quantified. Data are presented as the mean ± SEM (n=3). Statistical analysis was performed by Ordinary one-way ANOVA with symbols indicating p-value in comparison to WE. ns: not significant, *: $p<0.05$, **: $p<0.01$.
